# Toll-Like Receptor Agonist Augments Virus-Like Particle-Mediated Protection from Ebola Virus with Transient Immune Activation

**DOI:** 10.1371/journal.pone.0089735

**Published:** 2014-02-24

**Authors:** Karen A. O. Martins, Jesse T. Steffens, Sean A. van Tongeren, Jay B. Wells, Alison A. Bergeron, Samuel P. Dickson, John M. Dye, Andres M. Salazar, Sina Bavari

**Affiliations:** 1 Molecular and Translational Sciences, United States Army Medical Research Institute of Infectious Diseases, Fort Detrick, Maryland, United States of America; 2 Virology Division, United States Army Medical Research Institute of Infectious Diseases, Fort Detrick, Maryland, United States of America; 3 Office of Regulated Studies, United States Army Medical Research Institute of Infectious Diseases, Fort Detrick, Maryland, United States of America; 4 Oncovir Inc., Washington, DC, United States of America; Public Health Agency of Canada, Canada

## Abstract

Identifying safe and effective adjuvants is critical for the advanced development of protein-based vaccines. Pattern recognition receptor (PRR) agonists are increasingly being explored as potential adjuvants, but there is concern that the efficacy of these molecules may be dependent on potentially dangerous levels of non-specific immune activation. The filovirus virus-like particle (VLP) vaccine protects mice, guinea pigs, and nonhuman primates from viral challenge. In this study, we explored the impact of a stabilized dsRNA mimic, polyICLC, on VLP vaccination of C57BL/6 mice and Hartley guinea pigs. We show that at dose levels as low as 100 ng, the adjuvant increased the efficacy of the vaccine in mice. Antigen-specific, polyfunctional CD4 and CD8 T cell responses and antibody responses increased significantly upon inclusion of adjuvant. To determine whether the efficacy of polyICLC correlated with systemic immune activation, we examined serum cytokine levels and cellular activation in the draining lymph node. PolyICLC administration was associated with increases in TNFα, IL6, MCP1, MIP1α, KC, and MIP1β levels in the periphery and with the activation of dendritic cells (DCs), NK cells, and B cells. However, this activation resolved within 24 to 72 hours at efficacious adjuvant dose levels. These studies are the first to examine the polyICLC-induced enhancement of antigen-specific immune responses in the context of non-specific immune activation, and they provide a framework from which to consider adjuvant dose levels.

## Introduction

A variety of platforms currently exist for the development of viral vaccines. The platform selected can impact the balance between vaccine safety and efficacy. Attenuated viral vaccines and vaccines that utilize a vector-based expression system are highly immunogenic but raise concerns about safety and pre-existing immunity; DNA-based vaccines and protein-based vaccines, however, often lack the required immunogenicity for long term protection [1]–[3]. To augment the efficacy of protein-based vaccines, adjuvants that increase immunogenicity without causing deleterious, non-specific immune activation will be required.

The Ebola virus-like particle (VLP) vaccine has been shown to be highly efficacious in the mouse, guinea pig, and NHP models of filovirus infection [4]–[9]. VLP contain the viral matrix protein, VP40, and glycoprotein. Glycoprotein is the primary immunogenic component of the vaccine and is required for efficacy, while VP40 is required for particle formation [7], [10]–[13]. The expression of glycoprotein in a virus-like conformation is thought to increase the efficacy of the vaccine as it may permit the cross-linking of target receptors and concentrate the presence of antigen in antigen-presenting cells [14]. While the VLP is efficacious in the small animal models of filovirus infection without an adjuvant, inclusion of an adjuvant will provide valuable dose sparing and may enhance immunogenicity.

Alum-based compounds and a formulated monophosphoryl lipid A (MPL) are the only current FDA-approved adjuvants in human vaccines. However, the use of toll-like receptor (TLR) and other pattern-recognition receptor (PRR) agonists as adjuvants is a burgeoning area of study [15]–[18]. These molecules have the advantage of directly activating critical innate immune signaling pathways that could potentially enhance antigen-specific immune responses in a directed manner.

One particularly interesting TLR ligand that is being explored as a potential adjuvant is polyinosinic:polycytidylic acid (PolyIC). PolyIC is a dsRNA mimic that activates the innate immune response via TLR3 and the mitochondrial receptors MDA5 and possibly RIGI [19]–[21]. PolyICLC is a variant of polyIC that is stabilized by a poly-lysine chain. The poly-lysine chain prevents degradation of polyIC by serum nucleases that are present in primates, including humans [22]–[25]. While the induction of type I IFN by polyICLC has been harnessed for its therapeutic potential [26]–[32], polyICLC has also been shown to enhance antigen-specific T cell responses in HIV and malaria vaccines when administered as an adjuvant [33], [34]. It enhanced antigen-specific Th1 immune responses more significantly than TLR4, TLR7/8, or TLR9 agonists, and its efficacy is dependent on the triggering of type I interferon [21], [34]–[37]. However, there is concern that the level of non-specific immune activation that is required for adjuvant efficacy would be detrimental to the host.

In this study, we examine the ability of polyICLC to augment the protection afforded C57BL/6 mice and Hartley guinea pigs by Ebola VLPs. We characterize the antibody and T cell responses elicited by the VLP alone and show that the VLP is a highly efficacious vaccine that results in protection from Ebola virus challenge. We then demonstrate that the inclusion of polyICLC in the vaccine significantly increases anti-glycoprotein antibody titers and enhances T cell responses in mice. This is the first study to systematically compare the antigen-specific immune enhancement elicited by polyICLC to the levels of non-specific inflammation. We show that polyICLC provides dose sparing and immune enhancement at far lower adjuvant dose levels than those currently published in the literature, and the adjuvant-induced immune activation resolves rapidly. Together these data suggest that TLR3/MDA5 agonists can induce effective antigen-specific immune responses with only transient immune activation when complexed with a protein-based vaccine.

## Materials and Methods

### Ethics Statement

Research was conducted under an IACUC approved protocol in compliance with the Animal Welfare Act, PHS Policy, and other Federal statutes and regulations relating to animals and experiments involving animals. The IACUC committee approving this protocol is the United States Army Medical Research Institute of Infectious Diseases (USAMRIID) IACUC. The facility where this research was conducted, USAMRIID, is accredited by the Association for Assessment and Accreditation of Laboratory Animal Care, International and adheres to principles stated in the 8th Edition of the Guide for the Care and Use of Laboratory Animals, National Research Council, 2011.

### Animals, vaccinations, and viral challenge

C57BL/6 mice were obtained from NCI Charles River. Female mice between 8-12 weeks of age were vaccinated with 100 µl via the intramuscular (IM) route, in the caudal thigh. Female Hartley guinea pigs were obtained from Charles River and weighed between 400–800 g. Guinea pigs were vaccinated with 200 µl IM.

Animals were monitored at least once daily, and their status was evaluated according to an Intervention Scoresheet approved by USAMRIID IACUC. Monitoring increased to three times daily if the animals were given a score of three or four. Euthanization was by CO_2_ inhalation followed by confirmatory cervical dislocation. Analgesics and anaesthetics were not used in this study and animals were euthanized for humane purposes if they reached a score of five or more, which would be indicated if the animals exhibited ruffled fur, weakness, unresponsiveness, and/or difficulty walking. Otherwise, animals were euthanized on day 14 of the study. For all survival studies, control groups included animals vaccinated with saline and/or adjuvant alone.

PolyICLC (Hiltonol) was provided by Oncovir, Inc., and it was diluted in sterile saline for injection. MPL (MPLA-SM) was from Invivogen and R848 (Resiquimod) was from R&D Systems. VLPs were manufactured by Paragon Bioservices and were produced by transfecting 293T cells with Ebola Zaire virus glycoprotein and VP40, essentially as previously described [10]. VLP were irradiated at 1e6 rad to ensure sterility and contained less than 25 EU/ml endotoxin and less than 10 colony forming units (CFU) of bacteria per vaccination. VLP were diluted in sterile saline and combined with polyICLC prior to administration to animals. Vaccinations were administered IM two times, with three weeks between vaccinations. Viral challenge occurred four weeks after the second vaccination. A challenge dose of 1,000 pfu of mouse-adapted (ma-) or guinea pig (gp-) adapted Ebola Zaire virus was administered via the intraperitoneal route (IP) [38].

### Antibody Assays

Antibody titers were determined using an ELISA. Two µg/ml of recombinant Ebola virus glycoprotein was plated in a flat bottom 96 well plate overnight. Plates were incubated with blocking buffer (5% milk, 0.05% Tween in PBS) for 2 hours, and then serum samples were added to plates. The standard protocol used half log dilutions starting at a 1∶100 dilution; serum from polyICLC and VLP vaccinated mice was also tested at 1∶1 dilutions starting with a 1∶1000 dilution, to achieve increased granularity. After 2 hours, plates were washed with PBS+0.05% Tween and goat anti-mouse IgG-HRP secondary antibody was added at a 0.6 µg/ml. One hour later, plates were washed and exposed using Sure Blue TMB 1-component substrate and stop solution (KPL), and the absorbance at 450 nm was recorded. Serum from unvaccinated animals was used to establish background and titers were defined as the serum dilution resulting in an absorbance greater than 0.2, where background was universally less than 0.2. Serum from animals previously determined to contain anti-glycoprotein antibody was included in each assay to serve as a positive control.

### T cell assays – Intracellular cytokine staining

Mice were euthanized by CO_2_ inhalation at various time points after vaccination and splenocytes were harvested for T cell assays. For T cell cytokine assays conducted on the same day of splenocyte collection, cells were cultured at 10e6 cells/ml in complete media (45% RPMI 1640 + 45% Click’s EHAA media, 10% FBS, 20 mM Hepes, 1% Pen/strep, 0.05 mM BME) with 10 U/ml mouse recombinant IL2, 1 µg/ml mouse CD49d (BD #553314), 1 µg/ml mouse CD28 (BD 553295), and 1x protein transport inhibitor cocktail (eBioscience #00-4980). 1e6 cells were plated in each well of a 96 well plate and were stimulated with either PMA (50 ng/ml) and ionomycin (1 µg/ml) as a staining control, DMSO, Ebola virus peptides at 2 µg/ml, or Marburg peptides at 2 µg/ml, which did not elicit expansion of cells from Ebola VLP-vaccinated animals. Ebola virus glycoprotein peptide WIPYFGPAAEGIYTE was utilized for experimental samples, as it had previously been shown to elicit a T cell response in C57BL/6 mice [39], [40].

Six hours after stimulation, cells were washed in HBSS + 10% FBS. Live/dead aqua (Invitrogen) was used to identify viable cells by incubation for 20 minutes at 4C, and Fc Block (Miltenyi) was used to prevent non-specific antibody binding. After washing, surface antibodies (CD3 FITC clone 145-2C11, CD8 APC-H7 clone 53-6.7, CD4 PerCpCy5.5 clone RM4-5, from BD) were incubated with samples for 20’ at 4C, cells were washed again, and then Cytofix/Cytoperm was utilized as per the manufacturer’s instructions with IFNγ APC (clone XMG1.2), IL2 PECy7 (clone JES6-5H4), and TNFα PE (clone MP6-XT22) antibodies, all from BD. Cells were then run on the Canto II flow cytometer and analysis was conducted using FlowJo software. Isotype controls were used to define populations.

For cells that were cultured with recombinant Ebola virus glycoprotein prior to intracellular cytokine staining, splenocytes were incubated for six days with 0.1 µM Ebola Zaire virus glycoprotein as an experimental treatment, 0.1 µM Marburg Musoke virus glycoprotein, or ConA (1 µg/ml) as a proliferation control. Cells were cultured in complete media with 10 U/ml mouse recombinant IL2, 1 µg/ml mouse CD49d (BD #553314), and 1 µg/ml mouse CD28 (BD 553295). On days 2 and 4 of the stimulation, 10% of the T cell culture supplement without ConA (BD) was added to the media. On day 6, viable cells were isolated by ficoll gradient. Cells were then plated at 1e6/100 µl in 96 well plates and the intracellular cytokine assay described above was utilized.

### Measurement of serum cytokine levels

PolyICLC-induced cytokine and chemokine induction was measured using peripheral blood. Blood was collected in serum separators at various time points after vaccinations. Serum was diluted 1∶4 in PBS and evaluated using the BD Cytometric Bead Array Flex Sets for the following cytokines and chemokines: IL1α, IL1β, IL6, IL5, IL12p40, IFNγ, TNFα, MIP1α, MIP1β, MCP1, and KC. As a positive control, supernatant from cells cultured with PMA/ionomycin and LPS was utilized; all samples were run in duplicate. The assay was run according to the manufacturer’s instructions and the samples were evaluated on the BD FACS Canto II. IFNα and IFNβ levels were evaluated using an ELISA (PBL Interferon). Serum was diluted 1∶4 and samples were run in duplicate, according to the manufacturer’s instructions.

### Popliteal lymph node activation

Popliteal lymph nodes (LN) were isolated from vaccinated mice at various time points after vaccination. LN were filtered on a 70 µm filter and resuspended in HBSS. Cells were plated in 96 well plates and viable cells were identified using Invitrogen’s Live/Dead aqua dye. Surface stains examined B cell and NK cell activation (CD3 FITC clone 145-2C11, CD19 PE clone 1D3, CCR7 PerCpCy5.5 clone 4B12, CD86 PECy7 clone GL1, and CD69 APC clone H1.2F3 from BD, and NK1.1 APCH-7 clone PK136 from eBioscience) or innate cell activation, with a focus on dendritic cells (CD80 PE clone 16-10A1, CD11b PerCpCy5.5 clone M1/70, CD86 PECy7 clone GL1, CD11c APC clone HL3, CD8 APCH7 clone 53-6.7 from BD, and Class II FITC clone M5/114.15.2 from eBioscience). Fc Block (Miltenyi) was used to minimize non-specific antibody staining and isotype controls were used to set gates after gating on viable cells. Analysis was conducted on the BD FACS Canto II and with FlowJo.

### Statistical Analysis

The one-tailed Student’s T test was used to compare antibody titers, T cell responses, cytokine and chemokine levels, and cellular activation. P<0.05 was considered statistically significant. Survival studies were evaluated using Fisher's exact test with multiple testing corrections performed by permutation based on the number of comparisons performed.

## Results

### PolyICLC enhances the efficacy of VLPs

**Figure 1:**
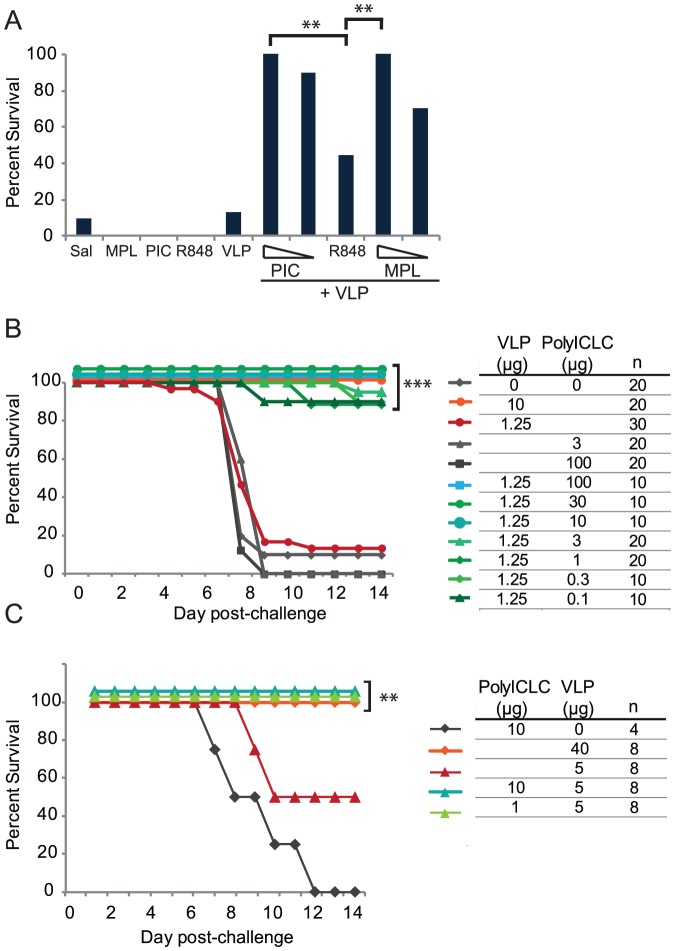
PolyICLC augments VLP-mediated protection from ma-EBOV and gp-adapted-EBOV challenge. C57BL/6 mice were vaccinated IM twice, three weeks apart, with 1.25 µg of VLP with or without 10 or 1 µg of polyICLC (PIC), 10 µg of R848, or 10 or 1 µg of MPL (A) or with dose levels of polyICLC ranging from 100 µg to 100 ng; “Sal” indicates Saline vaccination (B). Challenge occurred four weeks after the vaccine boost. (C) Hartley guinea pigs were vaccinated two times, three weeks apart, with 5 µg of VLP, with or without 10 µg or 1 µg of polyICLC. Nine or ten animals received R848 or MPL as adjuvant, respectively (A); polyICLC groups were repeated and the n is noted in (B). All p-values were calculated using Fisher's exact test with multiple testing corrections performed by permutation based on the number of comparisons performed. Significant comparisons between vaccinations with 10 or 1 µg of adjuvant (A) or between the low dose group (red lines for both A and B) and the low dose group in combination with polyICLC (B and C) are shown. “***” indicates p<0.0005 and “**” indicates p<0.005.

C57BL/6 mice were vaccinated with a suboptimal dose of VLP in combination with three adjuvants, polyICLC (a TLR3/MDA5 agonist), R848 (a TLR7/8 agonist), and MPL (a TLR4 agonist). Ten micrograms of polyICLC and MPL increased survival from 13% to 100%, while 10 µg of R848 increased protection to 44.4%. The enhanced protection afforded by 10 µg of R848 was statistically lower than that afforded by polyICLC or MPL. PolyICLC and MPL were therefore evaluated at a lower dose level, at which polyICLC was more effective (not significant) (Figure 1A).

Considering the efficacy of polyICLC at a dose level of 1 µg, we conducted a dose down using the suboptimal dose of VLP (1.25 µg) with 100 µg to 100 ng of polyICLC. Thirteen percent of animals vaccinated with 1.25 µg of VLP survived challenge. Inclusion of at least 100 ng of polyICLC resulted in survival of 90–100% of animals, which is a highly statistical increase in survival over vaccination with VLP alone (p<0.0001) (Figure 1B). A VLP dose level of 10 µg alone was sufficient to confer 90–100% protection in multiple iterations, and mice vaccinated with saline or with only polyICLC did not survive challenge.

The efficacy of polyICLC as an adjuvant was then tested in the guinea pig model of Ebola virus infection. Guinea pigs were vaccinated on the same schedule as mice and were challenged with gp-EBOV. All guinea pigs vaccinated with 40 µg of VLP were protected. Fifty percent of animals treated with 5 µg of VLP survived, but inclusion of either 10 or 1 µg of polyICLC increased survival to 100%, which was also a highly significant increase (p = 0.0048) (Figure 1C).

### PolyICLC enhances antibody titers of VLP-vaccinated mice and guinea pigs

**Figure 2:**
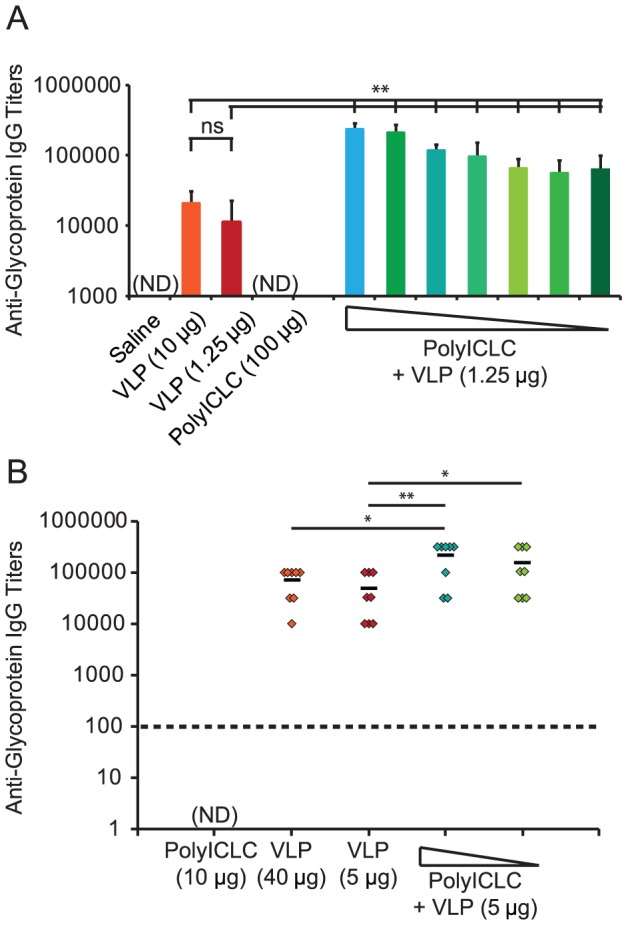
PolyICLC increases VLP-induced EBOV-specific antibody titers. (A) Peripheral blood was collected from the vaccinated C57BL/6 mice from Figure 1 two weeks after the vaccine boost. Anti-glycoprotein IgG titers in the serum were measured using recombinant glycoprotein as the antigen in an ELISA. (B) Peripheral blood was collected from the vaccinated Hartley guinea pigs from Figure 1 two weeks after the vaccine boost. Anti-glycoprotein IgG titers in the serum were measured using recombinant glycoprotein as the antigen in an ELISA. Dashed line indicates lower limit of detection of assay, which was 1∶100 for both A and B. The number of animals is the same as indicated in Figure 1. PolyIC:LC dose levels indicated by triangle are the same as those in Figure 1, and they range from 100 µg to 100 ng in the mouse vaccination studies and 10 µg to 1 µg in the guinea pig vaccination study. P values were determined using the Student’s T-test where “*” indicates p<0.05 and “**” indicates p<0.005. “ns” indicates “not significant”; “ND” indicated “not detectable”.

Two weeks after vaccinated C57BL/6 mice and Hartley guinea pigs received the second vaccination, peripheral blood was collected and evaluated for anti-glycoprotein antibody titers. PolyICLC inclusion increased anti-glycoprotein antibody titers in mice by over a log, and this increase was statistically significant both when compared to titers elicited by the 1.25 µg VLP dose without adjuvant and when compared to titers elicited by the 10 µg dose of VLP (Figure 2A). Anti-glycoprotein IgG titers decreased with decreasing dose levels of polyICLC, but they were consistently higher than the titers obtained with VLP vaccination alone.

Similar to what was observed in mice, vaccination with polyICLC enhanced anti-glycoprotein antibody titers in guinea pigs (Figure 2B). Titers in animals vaccinated with polyICLC were significantly higher than those observed in animals vaccinated with the 5 µg dose level of VLP.

### PolyICLC enhances antigen-specific T cell responses in mice vaccinated with Ebola VLP

Vaccination with 10 µg of Ebola VLP results in detectable, antigen-specific T cell responses, and these responses are predominantly detected in CD4 T cells. To determine whether polyICLC enhances antigen-specific T cell responses in VLP-vaccinated mice, we vaccinated mice two times with either 10 µg of VLP alone, 10 µg of VLP with 1 µg of polyICLC, or saline or polyICLC alone. Splenocytes were collected four weeks after the vaccine boost to identify antigen-specific T cell responses. A known T cell epitope (WIPYFGPAAEGIYTE) was used to identify antigen-specific responses [5], [39].

**Figure 3:**
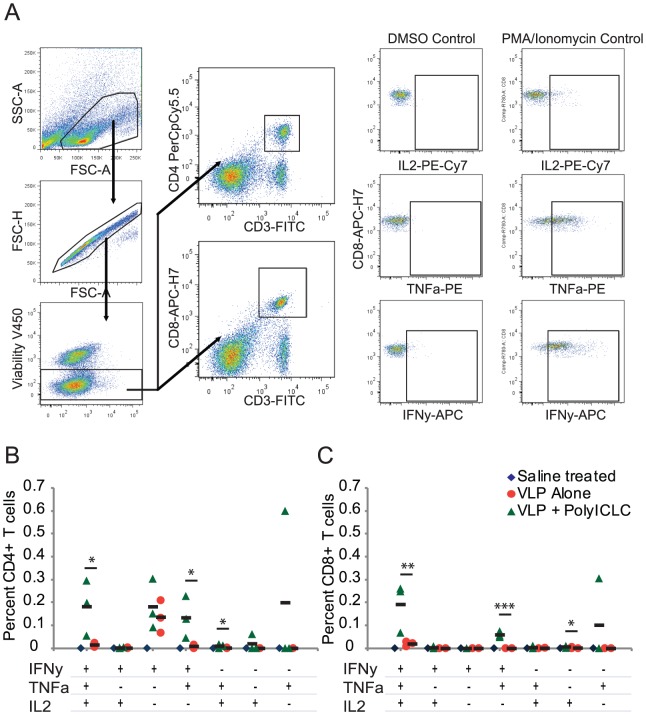
PolyICLC enhances antigen-specific T cell responses during VLP vaccination. C57BL/6 mice were vaccinated with 10 µg of VLP, with or without 1 µg of polyICLC. Vaccination was on the same schedule as in Figure 1, and control animals were vaccinated with saline or polyICLC alone. Four weeks after the vaccine boost, splenocytes were harvested and cells were re-stimulated with DMSO or Marburg peptides, PMA/ionomycin, or a known EBOV peptide. IFNγ, TNFα, and IL2 responses of viable CD4 and CD8 T cells were measured; gating was on lymphocytes, viable cells, and then CD3+CD4+ or CD3+CD8+ T cells (A). Percentages shown are the percent of the CD4+ (B) or CD8+ (C) T cell populations that were cytokine positive, after subtracting the background elicited by DMSO. P values were determined using the Student’s T-test by comparing the percentage of responding cells in animals vaccinated with VLP alone versus vaccination with VLP and polyICLC. “*” indicates p<0.05, “**” indicates p<0.005, and “***” indicates p<0.0005. Four animals were utilized in each group for each iteration of this experiment, and the experiment was repeated two times.

CD4 T cell responses, predominantly IFNγ-positive responses, were detected in animals vaccinated with VLP alone. The addition of polyICLC to the vaccine, however, greatly enhanced the frequency of polyfunctional CD4 T cell responses and resulted in detectable CD8 T cell responses (Figure 3). An average of 0.1825% of CD4 T cells from VLP + polyICLC vaccinated animals responded to glycoprotein peptide stimulation with an IFNγ+TNFα+IL2+ cytokine response; this is approximately ten-fold higher than the tri-functional response of cells from animals vaccinated with VLP alone. Polyfunctional CD8 T cell responses were also observed in animals vaccinated with VLP and polyICLC but not in animals vaccinated with VLP alone. PolyICLC increased both the frequency and functionality of antigen-specific T cells in vaccinated mice.

### PolyICLC enhances T cell responses in animals vaccinated with a suboptimal dose of VLP

While the enhancement of the T cell responses in animals vaccinated with a protective dose of VLP was promising, we were specifically interested in understanding whether polyICLC could augment VLP protection at a suboptimal vaccine dose. Mice were vaccinated with 1.25 µg of VLP, alone or in combination with 10, 1, or 0.1 µg of polyICLC. Seven days after the vaccine boost, splenocytes were harvested and subjected to the same intracellular cytokine assay and analysis described above. PolyICLC enhanced antigen-specific T cell responses in a dose-dependent manner, with a trend toward an increase in IFNγ response particularly apparent in the CD4 T cell population. The overall frequencies of responding cells in these vaccination groups, however, were quite low (data not shown).

Considering the low frequency of responding cells in these vaccination groups, we optimized a culture assay to examine the frequency of antigen-specific memory cell subsets in vaccinated animals. Animals received the same vaccination treatments as described above; additionally, a subset of animals were vaccinated with 10 µg of VLP, with or without 10 µg of polyICLC.

Twenty-eight days after the vaccine boost, splenocytes were harvested and cultured with 0.1 µM recombinant Ebola virus glycoprotein, ConA, or 0.1 µM recombinant Marburg virus glycoprotein. On the sixth day of culture, viable cells were isolated using a ficoll gradient, and they were then subjected to an intracellular cytokine assay.

**Figure 4:**
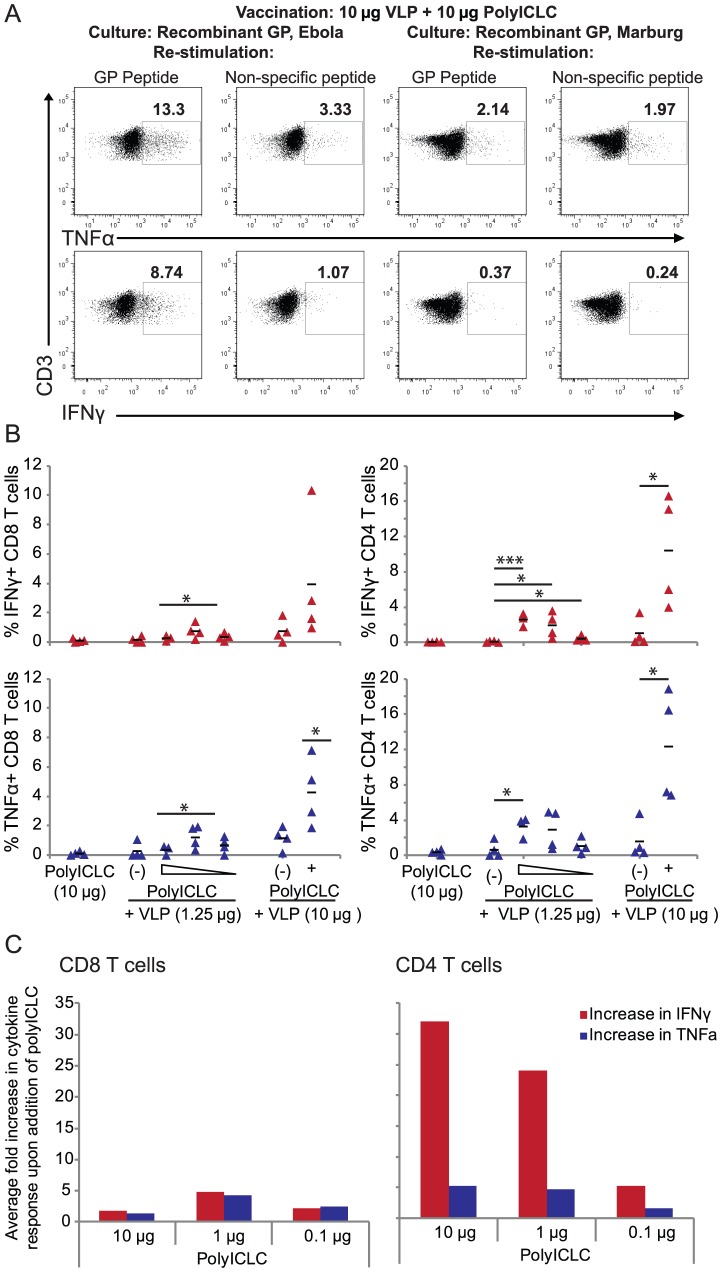
PolyICLC enhances antigen-specific T cell responses during VLP vaccination at suboptimal doses of antigen. C57BL/6 mice were vaccinated with polyICLC alone as a negative control, 10 µg of VLP, 10 µg of VLP with 10 µg of polyICLC, 1.25 µg of VLP, or 1.25 µg of VLP with 10, 1 or 0.1 µg of polyICLC. Three weeks later animals received a vaccine boost; four weeks later, animals were euthanized and splenocytes were collected. (A) Representative flow data are shown with CD3 on the y-axis and IFNγ or TNFα on the x-axis, after gating on lymphocytes, viable CD3+ cells, and CD4+ T cells. Data are from one representative animal vaccinated with the 10 µg of VLP + 10 µg of polyICLC, then cultured with either Ebola virus GP (left two panels) or Marburg virus GP (right two panels); viable cells were then re-stimulated with either GP peptide or non-specific peptides in the six hour intracellular assay. (B) Percentages of IFNγ (top) and TNFα (bottom) CD4 (right) and CD8 (left) T cells after re-stimulation of EBOV glycoprotein-cultured cells. Data shown are the percentages resulting from Ebola virus peptide stimulation after subtracting the percentages observed in cells re-stimulated with non-specific peptide. (C) Fold increase in IFNγ and TNFα positive cells after the addition of polyICLC to the 1.25 µg vaccination formulation. Fold difference shown for CD8 T cells (left) and CD4 T cells (right). Gating for B and C was on lymphocytes, viable cells, and then CD3+CD4+ or CD3+CD8+ T cells. P values were determined using the Student’s T-test by comparing the percentage of responding cells in animals vaccinated with VLP alone versus vaccination with VLP and various doses of polyICLC. “*” indicates p<0.05, “**” indicates p<0.005, and “***” indicates p<0.0005. Four to five animals were utilized in each group for each iteration of this experiment.

Cells from vaccinated animals that were cultured with recombinant Ebola virus glycoprotein responded to re-stimulation with the glycoprotein peptide (Figure 4A, left panels); re-stimulation with non-specific peptides did not elicit a response (Figure 4A, left center panels). Cells cultured with ConA or Marburg virus glycoprotein did not respond to re-stimulation with either the Ebola virus glycoprotein peptide or the non-specific peptide control (Figure 4A, right and right center). These data demonstrate that the cytokine response was specific to cells that were expanded in culture using Ebola virus glycoprotein.

The cytokine responses of viable cells cultured with Ebola virus glycoprotein and re-stimulated with Ebola virus peptide are shown in Figure 4B. The polyICLC-induced enhancement of the T cell response was primarily observed in CD4 T cells, though CD8 T cell responses were also detected. This bias toward a CD4 antigen-specific T cell response was comparable to that seen with freshly isolated cells, suggesting that the culture conditions did not impact the phenotype of the responding cells.

Figure 4C shows the fold increase in IFNγ and TNFα positive cells elicited by vaccination with VLP + polyICLC as compared to VLP alone. The increase was more significant in CD4 T cells than in CD8 T cells, and it was dependent on the dose level of polyICLC used. Within the CD4 T cell population, the frequency of cells responding with IFNγ was more significant than the increase in TNFα positive cells.

### PolyICLC elicits transient increases in serum cytokine and chemokine levels after vaccination

Having established that polyICLC can enhance the survival, antibody responses, and T cell responses of VLP vaccinated mice, we explored the impact of polyICLC on systemic immune activation. One concern with polyICLC has been its utility as an interferon-inducer; this aspect of polyICLC could raise concerns if it were used in a vaccine formulation.

To examine serum cytokine levels after polyICLC and VLP vaccination, C57BL/6 mice were vaccinated IM with various doses of VLP and polyICLC, alone or in combination. One hour, five hours, and twenty-four hours after vaccination peripheral blood was drawn via submandibular puncture, and cytokine and chemokine levels were evaluated.

**Figure 5:**
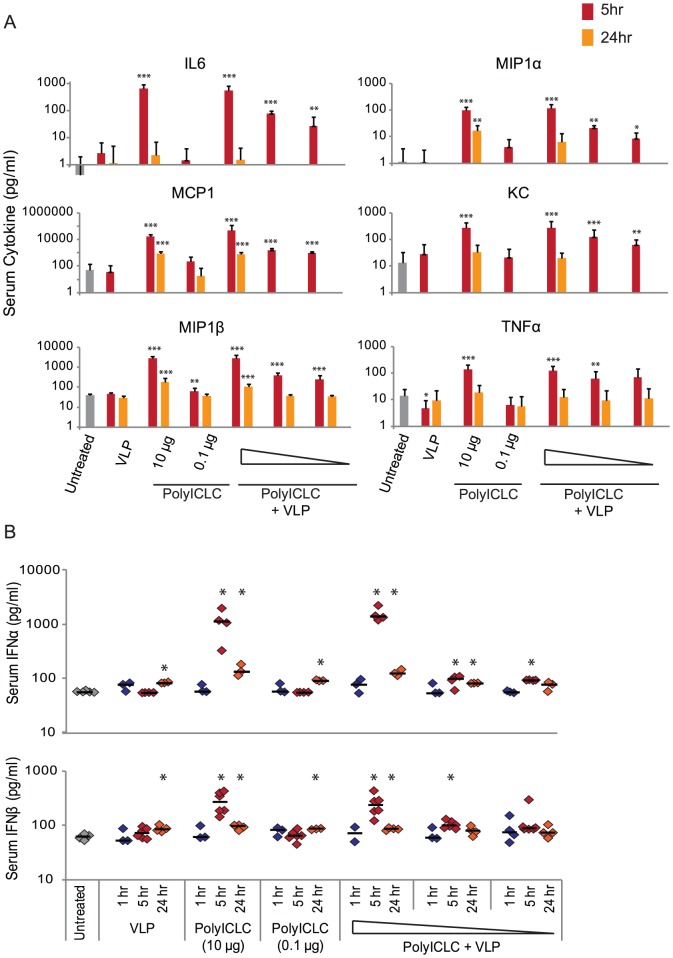
PolyICLC induces transient increases in serum cytokine and chemokine levels. C57BL/6 mice were vaccinated with 1.25 µg of VLP, 10 µg of polyICLC, 0.1 µg of polyICLC, or 1.25 µg of VLP with 10, 1, or 0.1 µg of polyICLC. One hour, five hours, or twenty-four hours after vaccination, peripheral blood was collected and evaluated for cytokine and chemokine levels. Supernatant from splenocytes stimulated with PMA/ionomycin or LPS served as a positive control to evaluate consistency between assays and to confirm the efficacy of cytokine and chemokine detection. (A) Six cytokine and chemokines that consistently increased in mouse serum after polyICLC or VLP (1.25 µg) treatment. Red bars indicate levels five hours after vaccination; orange bars indicate levels twenty-four hours after vaccination. The gray bar indicates the levels detected in untreated mice. Mean and standard deviation are shown. Triangle indicates decreasing dose levels of polyICLC (10 µg, 1 µg, 0.1 µg) administered in combination with 1.25 µg of VLP. (B) IFNα and IFNβ levels in the serum were detected using an ELISA. Median is indicated with the black bar. Triangle indicates decreasing dose levels of polyICLC (10 µg, 1 µg, 0.1 µg) administered in combination with 1.25 µg of VLP. The unpaired, two-tailed Mann-Whitney test was used to determine p-values; comparisons are between the indicated sample and the untreated control sample. “*” indicates p<0.05, “**” indicates p<0.005, and “***” indicates p<0.0005. Four to five animals were utilized in each group for each of the two iterations of this experiment, and only cytokines and chemokines showing consistent changes in expression between iterations are shown. Other cytokines and chemokines that were analyzed and which did increase in the control sample but did not increase after experimental treatment include the following: IL12p40, IFNγ, IL5, IL1α, and IL1β.

Vaccination with 1.25 µg of VLP did not impact serum cytokine and chemokine levels in the serum, but treatment with polyICLC increased the level of eight cytokines and chemokines. The cytokines and chemokines showing the greatest increase after vaccination are shown in Figures 5A and 5B at both five hours and twenty-four hours after vaccination; no significant increases in any cytokine or chemokine were observed one hour after vaccination. The cytokines and chemokines induced by polyICLC treatment included MIP1α (CCL3), MIP1β (CCL4), MCP1 (CCL2), KC (CXCL1), IL6, and TNFα (Figure 5A), as well as IFNα and IFNβ (Figure 5B).

Increases in cytokine and chemokine levels were resolved within twenty-four hours of treatment in mice administered 1 µg or 0.1 µg of polyICLC. Animals treated with 10 µg of polyICLC had significantly higher levels of MIP1α, MIP1β, IFNα, IFNβ, and MCP1 24 hours after vaccination in comparison to before vaccination, but these levels were decreased compared to the five hour time point. In general, therefore, increases in serum cytokine levels resolved or were in the process of resolving within twenty-four hours of treatment. Vaccination with VLP in combination with polyICLC did not impact the cytokine and chemokine levels that were elicited by polyICLC alone.

### PolyICLC activates cells of the draining lymph node in a dose-dependent manner

Having determined that increases in cytokine and chemokine levels were transient after polyICLC treatment, we next examined the draining lymph nodes of vaccinated mice to determine the persistence of immune activation after polyICLC injection. Mice received an IM injection of 10, 1, or 0.1 µg of polyICLC. The popliteal lymph node was then harvested, and cells were stained for various innate immune cell markers and activation markers to track changes in immune cell activation after polyICLC treatment. The focus was primarily on dendritic cells (DC) and natural killer (NK) cells, as these have been previously implicated in polyICLC-induced activation in vitro [21]. The general CD11b+CD11c– population, which includes macrophages, neutrophils, and eosinophils, was also examined for up-regulation of co-stimulatory molecules, as were B cells (CD19+CD3–).

**Figure 6:**
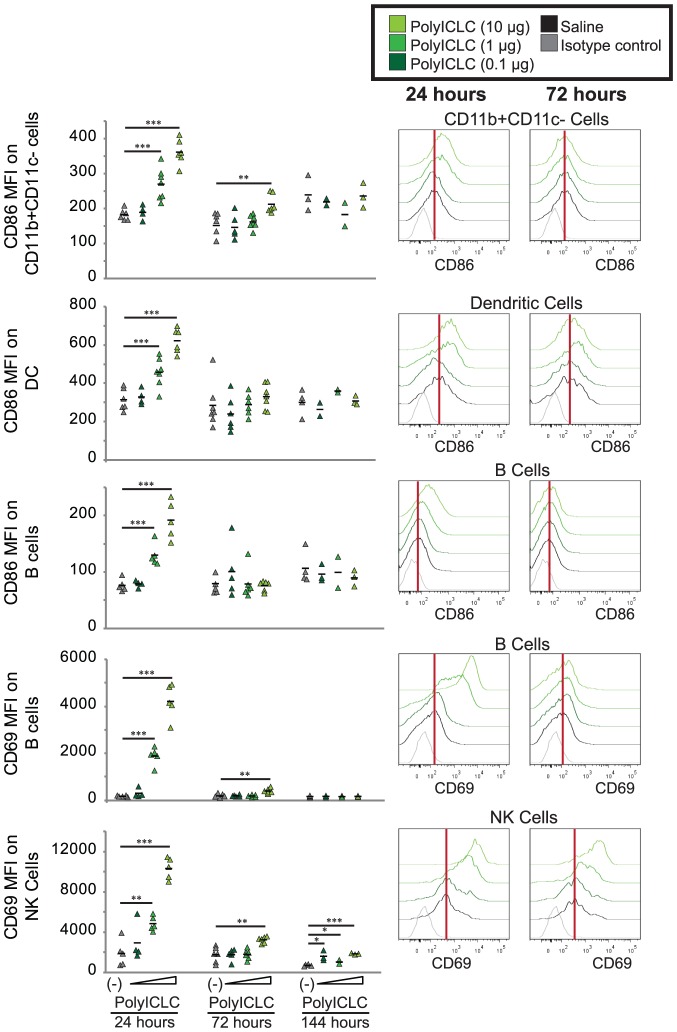
PolyICLC induces transient activation of cells in the draining lymph node. C57BL/6 mice were vaccinated IM with saline or 0.1 µg, 1 µg, or 10 µg of polyICLC (dose level indicated by triangle), and the popliteal lymph node was collected 24, 48, or 144 hours after vaccination. Viable cells were stained for common innate immune markers as well as CD19 for B cells and NK1.1 for NK cells. The mean fluorescence intensity (MFI) of CD69 and CD86 were used to evaluate the activation of appropriate cell populations. Dot plots to the right indicate gating of the parent cell after gating on viable lymphocytes. Histograms show CD86 and CD69 expression on the parent cells 24 hours and 72 hours after vaccination. 200,000–500,000 events were collected in duplicate for each animal. P values were determined using the Student’s T-test by comparing the percentage of responding cells in animals vaccinated with VLP alone versus vaccination with VLP and various doses of polyICLC. “*” indicates p<0.05, “**” indicates p<0.005, and “***” indicates p<0.0005. Four to five animals were utilized in each group for each iteration of this experiment.

Twenty-four hours after vaccination, activation was apparent in CD11b+CD11c– cells, CD11c+ dendritic cells, B cells, and NK cells of the draining lymph node (Figure 6). Activation, as determined by the mean fluorescence intensity of CD86 and CD69, increased in a dose-dependent manner. Activation in cells from animals treated with 0.1 µg of polyICLC did not differ from animals receiving a saline injection, but cells from animals treated with 1 µg or 10 µg of polyICLC exhibited significantly elevated cellular activation.

To explore the persistence of localized immune activation, we sampled lymph nodes 72 hours and 144 hours after treatment. Activation following the injection of 1 µg of polyICLC resolved within 72 hours; activation following the injection of 10 µg of polyICLC was still significantly higher than that seen in saline-treated animals at 72 hours, but resolved within 6 days of treatment. NK cell activation was statistically different from saline treated animals at day 6, but was less than that observed at 72 hours.

**Figure 7:**
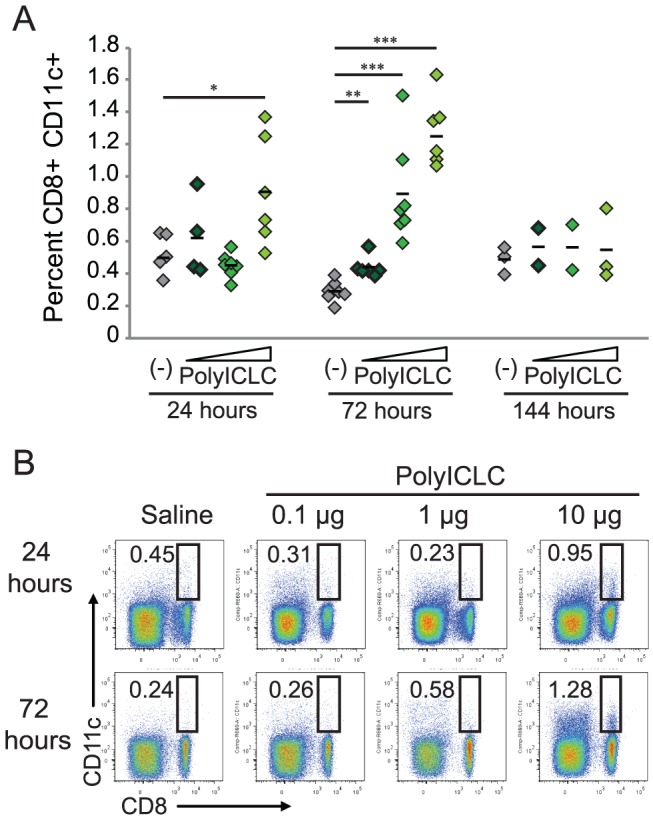
PolyICLC results in an increase of CD8α+ DC to the draining lymph node. (A) The percentage of CD8α DC in the popliteal lymph node after vaccination, as described in Figure 6. (B) CD8α DC percentages are the percentages of viable lymphocytes collected from the popliteal lymph node. Dot plots show CD11c on the y-axis and CD8α on the x-axis with samples from animals treated with saline, 0.1, 1, or 10 µg of polyICLC (dose level indicated by triangle) 24 hours or 72 hours before sample collection. 200,000–500,000 events were collected in duplicate for each animal. P values were determined using the Student’s T-test by comparing the percentage of responding cells in animals vaccinated with VLP alone versus vaccination with VLP and various doses of polyICLC. “*” indicates p<0.05, “**” indicates p<0.005, and “***” indicates p<0.0005. Four to five animals were utilized in each group for each iteration of this experiment.

In addition to cellular activation, we also noted an increase in the presence of CD8α DC in the draining lymph node after vaccination (Figure 7). An influx of cells was apparent within 24 hours in animals treated with 10 µg of polyICLC, but was significant in all treated groups at 72 hours after treatment. This increase in CD8α DC was resolved by the final sample day.

## Discussion

The VLP vaccine is highly efficacious against Ebola and Marburg viral challenge. The vaccine has successfully protected NHP from viral challenge via both the aerosol and IM routes of infection, and it has been used in combination with several adjuvants, including QS21 and RIBI ([8], [9] and unpublished data). As a protein-based vaccine, the filovirus VLP is an excellent model from which to evaluate the balance between safety and immunogenicity of potential adjuvants. In multiple studies comparing the potential role of TLR agonists as adjuvants, polyIC and its analogs have elicited more robust immune responses than ligands for other TLR receptors [34], [36], [37]. These data were corroborated by our survival studies, which compared polyICLC to TLR4 and TLR7/8 agonists. We therefore selected the TLR3 and MDA5 ligand polyICLC for our studies.

Vaccination with VLP induces antigen-specific antibody titers, which include neutralizing antibodies, as well as both CD4 and CD8 T cell responses [5], [11]. While the precise correlates of vaccine-mediated protection from filoviruses have not been defined, there is a critical role for high levels of antigen-specific antibody. The requirement specifically for anti-glycoprotein antibody has been observed in several models of filovirus infection, though the function of this antibody is not well-established: there is no clear link between the emergence of neutralizing antibody and protection [41], [42]. Polyfunctional T cell responses are also associated with effective vaccination in several vaccine models and are thought to be required for antiviral immunity; they were therefore examined in these studies [41], [43]–[46].

The addition of polyICLC to the VLP enhanced antibody titers and CD4 and CD8 T cell responses, and it increased the frequency of polyfunctional T cell responses. The increase in CD8 T cell responses was particularly striking: CD8 T cell responses were undetectable in animals vaccinated with VLP alone, but the inclusion of polyICLC resulted in antigen-specific, IFNγ+TNFα+IL2+ CD8 T cells. Protein-based vaccines typically elicit an antibody and CD4 T cell immune response rather than a CD8 T cell response. An IFN-inducing adjuvant like polyICLC may increase cross-presentation of antigen, resulting in stronger CD8 T cell responses and in increased efficacy against viral pathogens [47]. The association between polyICLC and cross-presentation was supported by the increase in CD8α DC in the draining lymph nodes of mice treated with polyICLC. This particular subtype of DC is associated with antigen cross-presentation and the emergence of a Th1 immune response, which likely augments antigen-specific T cell responses [48]–[50]. CD8α+DEC–205+ DC produce type I IFN in vitro upon stimulation with polyICLC [21]; our ex vivo data corroborate a role for CD8α DC in polyICLC immunogenicity.

Cumulatively, our data show that polyICLC is effective when used as an adjuvant in terms of immune enhancement and dose sparing. A critical aspect of adjuvant selection, however, involves safety. High levels of non-specific immune activation can lead to immune cell exhaustion, and immune activation can negatively impact individuals with autoimmune disorders or chronic infections [51]. A recent study examined subcutaneous injection of 1.6 mg of polyICLC into human volunteers and documented the presence of mild to moderate side effects that included flu-like symptoms and an immune profile mirroring that of a virally-infected individual [52]. These observations are promising in that a viral mimic holds the best possibility of stimulating an effective immune response; identifying an appropriate balance between dose level and efficacy, however, is of great import.

Previous studies utilizing polyIC or polyICLC as an adjuvant have used dose levels several logs higher than those described in our report. In murine vaccination models, dose levels of 50 µg (administered subcutaneously) were administered with a malaria vaccine [33], [53]. A liposome-encapsulated form of polyICLC was also tested with intranasal administration in mice, wherein a dose of 20 µg of polyICLC increased the efficacy of an H5N1 vaccine [54]. In NHP, our group has utilized 100 µg of polyICLC as an adjuvant with the VLP with great success [55], but 1 to 3 mg of polyICLC or polyIC is commonly used in NHP vaccination studies [24], [33]–[35].

To explore the relationship between adjuvant efficacy and toxicity, we examined the elevation of serum cytokine and chemokine levels after polyICLC vaccination. Serum cytokine and chemokine profiles are frequently used as a marker of toxicity [56]. We had anticipated an induction of IL12p40 and a high induction of type I IFNs after polyICLC injection. Increases in type I IFNs were transient and IL12p40 levels did not increase after treatment with 10 µg of polyICLC, though we observed that IM treatment with 50 µg of polyICLC did elicit circulating IL12p40 (unpublished observations). TNFα, IL6, IFNα, and IFNβ were elevated after treatment with 1 or 10 µg of polyICLC, similar to observations after murine IP treatment [21], and we further document up-regulation of the chemokines MCP1, MIP1α, KC, and MIP1β after adjuvant injection. Cytokine and chemokine levels peaked around five hours post-treatment, resolving twenty-four hours after treatment. While MCP1, MIP1α, and MIP1β are broadly associated with recruitment of circulating immune cells to the site of trauma, KC is specifically associated with neutrophil recruitment, suggesting broad immune cell recruitment following polyICLC administration. IL6, MCP-1, and TNFα have been defined by one author as being involved in “systemic inflammatory response syndrome”, while MIP1 and TNFα are more specifically associated with “neutophilic inflammation” [56]. Maintaining only a transient increase in these biomarkers after treatment may be important in terms of minimizing vaccine toxicity.

It has previously been shown that polyICLC can activate innate immune cells in vitro [21], but ex vivo examination of draining lymph nodes had not been conducted, to our knowledge. We measured activation 24, 72, and 144 hours after vaccination using three different dose levels of polyICLC, IM. CD11b+CD11c- cells, dendritic cells, B cells, and NK cells all exhibited transient immune activation after treatment and activation was dose dependent. Within 72 hours, activation was resolved in all but the highest polyICLC dose group, and activation in animals treated with 10 µg of the adjuvant resolved within 144 hours. The observation of activation in CD11b+CD11c- and CD11c positive cells, as well as in NK cells and B cells, suggests broad immune cell recruitment after polyICLC treatment.

PolyICLC has previously been tested in human clinical trials as a cancer therapeutic. Glioma regression was augmented after administration of 10–50 µg/kg of polyICLC with few side effects [57], [58], but the majority of clinical trials with polyICLC have utilized significantly higher dose levels [26], [28], [59]. Adapting polyICLC or other PRR agonists for use as vaccine adjuvants will require a better understanding of the relationship between side effects, inflammation, and efficacy. While it is clear that polyICLC enhances the immune response upon vaccination, this study is the first to evaluate the impact of polyICLC in the context of safety and toxicity. The data presented here suggest that optimizing adjuvant dosage can result in an efficacious protein-based vaccine formulation with minimal long term inflammation. Further research to define the relationship between dose levels of inflammatory agents in rodents with dose levels in humans would be of great utility.

### Disclaimer

Opinions, interpretations, conclusions, and recommendations are those of the author and are not necessarily endorsed by the U.S. Army.
